# Understanding Participant (Dis)Engagement From Domestic Violence Perpetrator Group Programs: A Review and Qualitative Synthesis

**DOI:** 10.1177/15248380251381824

**Published:** 2025-11-12

**Authors:** Brittany Ralph, Nicola Helps, Jasmine McGowan, Kate Fitz-Gibbon

**Affiliations:** 1 Monash University, Clayton, VIC, Australia; 2 University of Liverpool, UK; 3 Australian’s National Research Organization for Women’s Safety, Sydney, NSW, Australia; 4 University of Melbourne, Parkville, VIC, Australia

**Keywords:** domestic violence, perpetrator programs, intervention, completion, engagement

## Abstract

Men’s behavior change programs are a crucial component of contemporary efforts to address domestic violence, particularly (though not exclusively) across the Global North. Yet studies of program effectiveness consistently report high attrition rates and, importantly, an association between non-completion and recidivism. Scholars are thus increasingly concerned with understanding factors that predict program completion. However, limited research examines engagement beyond this binary notion of completion/non-completion. Adopting a broader scope, our systematic review examined English language, peer-reviewed research into the risk and protective factors that may influence engagement with/disengagement from all-male domestic violence perpetrator group programs. Our review was conducted in accordance with the Preferred Reporting Items for Systematic Reviews and Meta-Analyses (PRISMA) statement. Databases searched include ProQuest, Ovid, EBSCOhost, and Web of Science, from which 164 sources were subject to full-text review, and 80 sources (published 1988–2023) were included. The sources report on studies that use quantitative, qualitative, and mixed methods research designs, based in the United States, Canada, the United Kingdom, Australia, Portugal, and Spain. Through narrative synthesis, we explore how this research conceptualizes completion, considers (or indeed overlooks) engagement, and makes sense of risk/protective factors for non-completion and disengagement. Our findings highlight the inconsistent and often arbitrary definition and measurement of program completion across studies, calling into question its usefulness as a proxy for program effectiveness. Most crucially, our findings support the need for more nuanced analyses of program outcomes that capture the non-linear, relational process with which people engage in perpetrator programs, and behavior change more broadly.

## Introduction

In efforts to address domestic violence, scholars and policymakers across the globe are increasingly concerned with holding men accountable for their use of violence in ways that also meaningfully engage them in long-term behavior change. Behavior change programs for people who use domestic violence have thus emerged as a crucial intervention in many Western countries (Babcock et al., 2016). Within this context, a growing body of research examines program effectiveness, predominantly through recidivism data (Arce et al., 2020; Arias et al., 2013; Babcock et al., 2004; Feder & Wilson, 2005; Karakurt et al., 2019; Travers et al., 2021). Existing international reviews of this literature show some reduction in reported violence post-treatment (Arias et al., 2013; Babcock et al., 2004; Karakurt et al., 2019), though studies often conclude that the evidence on effectiveness is mixed. More recently, scholars have turned their attention to what drives divergent program outcomes, primarily seeking to measure factors that predict “completion” (Jewell & Wormith, 2010; Rondeau et al., 2001), and often reporting a correlation between non-completion and recidivism (Bennett et al., 2007; Hendricks et al., 2006; Olver et al., 2011).

Yet claims of an association between completion and recidivism have limitations as they often draw on official records, such as re-arrest data, that present a partial picture of violence (Arce et al., 2020; Feder & Wilson, 2005). Thus, reported reductions in the use of violence are “not sufficient to determine the real rates of recidivism” and should be read with caution (Arce et al., 2020, p. 159; Feder & Wilson, 2005). Victim-survivor accounts can provide crucial insights into violence not captured in official records and, when utilized in place of recidivism records, often indicate less positive program outcomes (Arce et al., 2020; Feder & Wilson, 2005). The value of alternative data sources to cross-validate perpetrators’ self-reports of change is thus increasingly recognized (McGinn et al., 2016; Helps et al., 2026; Vall et al., 2023; Westwood et al., 2020). However, the duty to effectively assess behavior change and evaluate programs cannot rest solely on the shoulders of victim-survivors.

There is thus a need for greater nuance in examining program outcomes and effectiveness. Looking beyond official records to assess the use of abusive behaviors is one crucial part of this conversation, another is the examination of how program “completion” is measured and whether focusing on an effective measurement of “engagement” is more pertinent to discussions about program efficacy (Fitz-Gibbon et al., 2024). There is variation in how programs define and track completion, and concerns as to how meaningful an assessment of completion may be, given participants may technically complete without taking on board learnings, accepting accountability for their use of violence, or working toward changing their behavior (see further, Fitz-Gibbon et al., 2024). Several reviews examine program effectiveness (Olver et al., 2011), including completion and recidivism, yet no known reviews examine the state of knowledge on the more nuanced and multi-dimensional notion of program *engagement*.

### The Current Study

This article reports the findings of a systematic review that examines how research into domestic violence perpetrator programs conceptualizes completion, considers (or indeed overlooks) engagement, and makes sense of risk/protective factors for non-completion and disengagement. The findings are structured in three parts. First, we explore definitions and measurement of completion, highlighting both inconsistency and a lack of justification across the studies and the interventions they examine. Second, we examine the breadth of risk and protective factors that have been found to impact program completion, and illustrate the complex and at times conflicting results that emerge from these types of analyses. Noting that several reviews aggregate predictors of program completion (Daly & Pelowski, 2000; Jewell & Wormight, 2010; Olver et al., 2011), our purpose is not to repeat these studies. Rather, we contribute to the broader academic and policy debate about assessing program effectiveness in this field. To this end, in the third findings section, we explore studies that offer a nuanced definition, operationalization, and discussion of engagement beyond completion. Our findings highlight the need for programs (and their evaluators) to move beyond a binary model of completion/non-completion, and take up more in-depth, qualitative research on the complexity of men’s engagement in perpetrator programs.

## Method

### Search Strategy

This systematic review followed the Preferred Reporting Items for Systematic Reviews and Meta-Analyses (PRISMA) statement (Moher et al., 2009). A protocol was registered on PROSPERO (PROSPERO 2023 CRD42023399763). The following databases/platforms were searched: ProQuest (Criminology Collection, International Bibliography of the Social Sciences, Social Science Database, Sociology Collection), Ovid (PsycINFO, Social Work Abstracts), EBSCOhost (Criminal Justice Abstracts, Violence and Abuse, and Cumulative Index to Nursing and Allied Health Literature [CINAHL Plus]), and Web of Science. The authors developed the search strategy in consultation with a university subject librarian. As outlined in Table 1, the search terms used were broad. Petticrew and Roberts (2008, p. 84) note that this is not uncommon in the social sciences where “less specific” and “more exhaustive” search terms are often required to identify relevant sources.

**Table 1.** Search Strings.

String 1	((intimate OR partner* OR domestic OR Family) AND (violence OR abus*)) OR batterer* OR non-violence
String 2	(men OR male OR men’s OR perpetrator* OR batterer* OR non-violence) Proximity-5 (program* OR behavio?r change OR treatment* OR intervention*)
String 3	engageme* OR participat* OR attend* OR complet* OR drop-out OR attrition OR complian* OR outcome*

Search results were imported into Covidence^1^ software for screening. As completion and engagement were often examined within or as biproducts of other research objectives (predominantly program effectiveness), articles were included for full-text review if they met the inclusion criteria or were deemed *potentially* relevant. Initially, 100 sources (title and abstracts) were screened by three authors (BR, NH and JM) to identify any discrepancies in the application of the criteria. The remaining abstracts were then screened by either BR, NH and/or JM, with all titles and abstracts screened by two authors independently. This process was repeated for full papers. At each stage, conflicts were reviewed and discussed by BR, NH and JM until consensus was reached.

### Inclusion and Exclusion Criteria

Qualitative, quantitative, and mixed-methods studies were included. Due to time and resource constraints, only English-language journal articles that reported findings of primary studies were included, and books, book sections, methods papers, theses, conference papers, and gray literature were excluded. No restrictions were placed on publication year. The population of interest was participants in all-male domestic violence perpetrator group programs, both mandated and voluntary, and in both correctional and community settings. Combined treatment models (e.g., interventions that address domestic violence *and* alcohol and other drug [AOD] use) were included if addressing domestic violence perpetration was a core component of the intervention. Other interventions, including one-to-one counseling, couples therapy, conjoint group therapy, offender programs that are not specific to domestic violence, and interventions for victim-survivors, were excluded. We applied the inclusion criteria with some leniency (being guided primarily by how authors presented their aims), to capture a broad range of literature on program completion and engagement. We acknowledge that this leniency allows subjectivity to influence our dataset. Discussion and debate between authors were thus encouraged, and decisions required consensus. There is substantial variation in the nature and extent to which completion and/or engagement are explored across the 80 included sources, which we believe adds nuance, thus aligning with our broader purpose.

### Data Extraction

BR and NH extracted data from each source, including: citation; year and location of study; intervention details including theoretical framework, program length, and delivery format; completion rate; outcomes influencing completion such as participant demographics, AOD assessments; definition of completion; and outcomes related to engagement. To ensure consistency, both authors completed data extraction for 10% of the sources (*n* = 8), and then cross-checked their results. Discrepancies were resolved by consensus between BR and NH.

### Quality Appraisal

Quality appraisal was performed by BR and NH using three tools for quantitative (Law et al., 1998), qualitative (Lockwood et al., 2015), and mixed-methods (Hong et al., 2018) studies. Each source was assessed by one author, however, 10% (*n* = 8) were assessed by two authors and cross-checked for consistency. The appraisal score represents the proportion of “yes” responses to the overall criteria count, with “not reported” regarded as a “no” response. The scores ranged from 0.4 to 1.0, averaging 0.72 (*M, SD* = 0.14). Of the 80 included sources, 4 (5.0%) were appraised as low quality (<0.5), 45 (56.25%) sources scored 0.5 to 0.74, and 30 (37.5%) sources were considered high-quality scoring >0.75. Quality was not an inclusion criterion for this study as our review sought to explore the *nature* of research into completion, rather than aggregate its key findings.

## Results

The initial database search resulted in 4,076 citations, as shown in Figure 1. After duplicates (*n* = 2,477) were removed, titles and abstracts (*n* = 1,599) were screened according to the inclusion criteria, resulting in 164 sources for full-text review. Eighty sources were included for data extraction and analysis. Table 2 provides details on the included sources.

**Figure 1.**
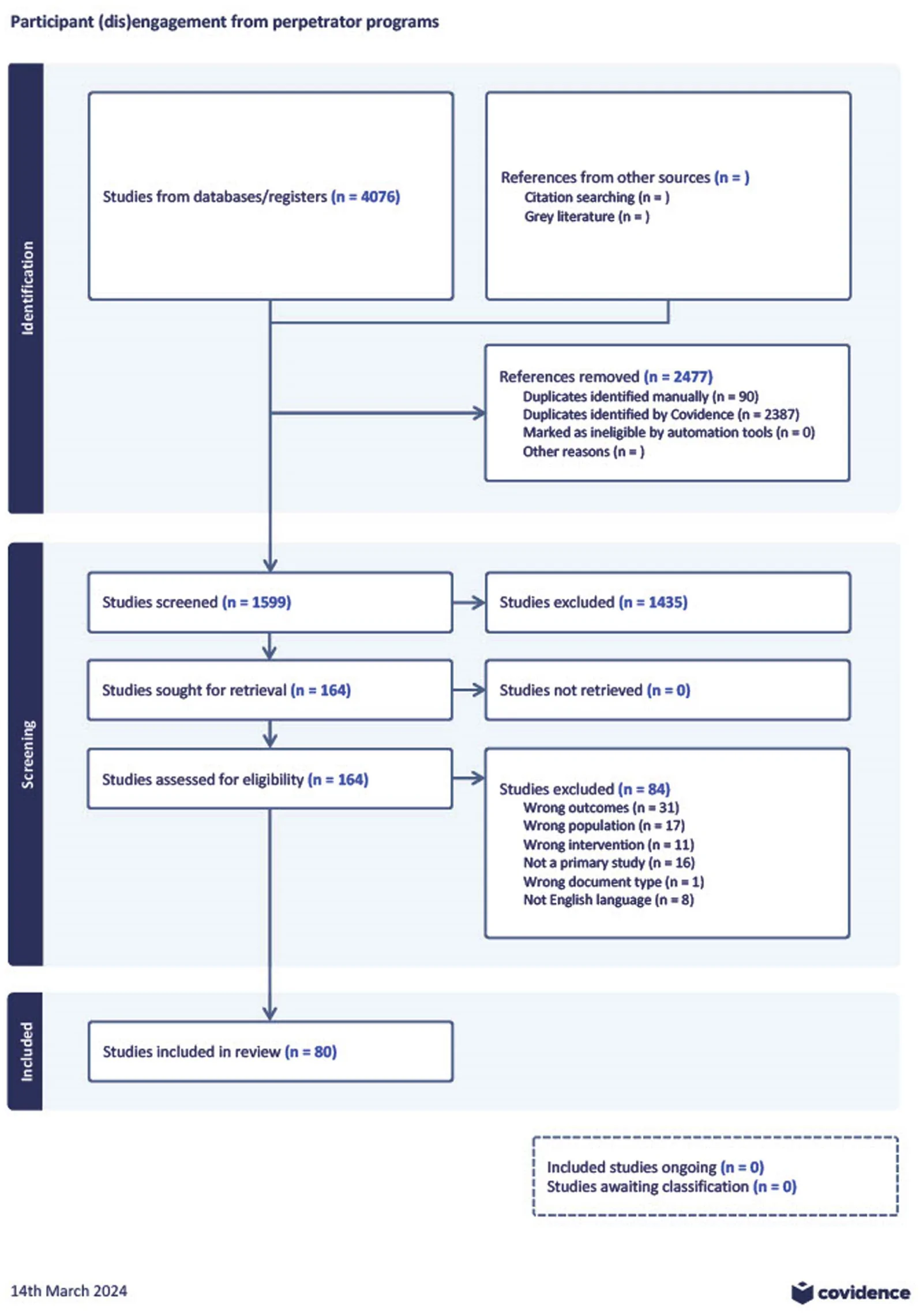
PRISMA diagram (generated using Covidence^1^).

**Table 2.** Summary Details of Included Studies.

Author (Year); Country	Method (Sample)	Intervention Approach	Program Length	Definition of “Completion”	Overall Completion Rate	AFM Data	Engagement Themes
Bellini and Westmarl (2021); USA	Qual (*n* = 6)	Duluth (adaptation pilot)	27 Weekly 90-min sessions	Not reported	Not reported	No	Groupwork, participation, conduct
Bennett et al. (2010); USA	Quant (*n* = 540)	Not reported	Not reported	Active participation, non-violence skills, program requirements, referral compliance	76.4% (*n* = 413)	No	Active participation, use of non-violence skills
Bouchard and Wong (2021); Canada	Qual (*n* = 180)	12 programs, multiple approaches (including, Duluth and cognitive-behavioral therapy [CBT])	Multiple treatment lengths, none reported.	Multiple definitions of completion, none reported.	Not reported	No	Men’s perspectives on group cohesion, therapeutic alliance, program logistics
Bowen and Gilchrist (2006); UK	Quant (*n* = 120)	Duluth-informed pro-feminist psycho-educational group format	1 × induction, 24 × 2.5-hr group sessions, and 5 × 2.5 hr follow-up sessions (11 months).	Could miss three sessions, but program staff defined participants as completers	67.5% (*n* = 81)	No	N/A
Brekke (1989); USA	Quant (*n* = 12)	CBT, structured skills group	Unclear (stated as both 14 and 20 weeks)	Not reported	87.5% (*n* = 9)	No	N/A
Brodeur et al. (2008); Canada	Quant (*n* = 202)	Five programs, no detail provided	Five programs, no detail provided	Five programs (definition of completion varied)	43.1% (*n* = 87)	No	N/A
Burnette et al. (2017); USA	Quant (*n* = 108)	CBT	26 weekly 2-hr sessions	All sessions	75.7% (*n* = 84)	No	N/A
Buttell and Carney (2002); USA	Quant (*n* = 137)	CBT	16 weekly 2-hr sessions	All sessions	56.2% (*n* = 77)	No	N/A
Buttell and Carney (2008); USA	Quant (*n* = 1,702)	CBT	26 weekly 2-hr sessions	All sessions	Not reported	No	N/A
Buttell and Pike (2002); USA	Quant (*n* = 91)	CBT	12 weekly 2-hr sessions	All sessions	72.5% (*n* = 66)	No	N/A
Cantos et al. (2015); USA	Quant (*n* = 456)	Multiple programs, most Duluth and CBT informed	Multiple programs, no detail provided	Min. 24 weeks of treatment plus other standards	53.6% (*n* = 218)	No	N/A
Carbajosa et al. (2017); Spain	Quant (*n* = 210)	Psycho-educational	32 Sessions	Participants who missed 2+ sessions considered dropouts.	77.1% (*n* = 162)	No	N/A
Carney et al. (2006); USA	Quant (*n* = 114)	CBT	16 weekly 2-hr sessions	All sessions	Not reported	No	N/A
Catlett et al. (2010); USA	Quant (*n* = 154)	Multiple programs (psycho-educational, therapeutic and didactic/confrontational)	16 weekly 90-min sessions	Could miss two sessions	54.5% (*n* = 84)	No	N/A
Chang and Saunders (2002); USA	Quant (*n* = 218)	Program 1: CBT and feminist; Program 2 process-psychodynamic	20 weekly 2.5-hr sessions	Could miss four sessions	62% (*n* = 134)	No	N/A
Chovanec (2012); USA	Quant (*n* = 95)	Psycho-educational	18 weekly 3-hr sessions	Not reported	55% (*n* = 52)	No	Groupwork (GEM-27)
Chovanec (2014); USA	Qual (*n* = 14)	Program 1: psycho-educational, Program 2: education-focused	P1: 18 weekly 3-hr sessions, P2: 23 weeks	N/A	P1: 50% (*n* = 13) P2: 32% (*n* = 9)	No	Men’s perspectives on program engagement
Chovanec and Roseborough (2017); USA	Quant (*n* = 81)	Psycho-educational and Duluth	18 weekly 3-hr sessions	Not reported	Not reported	No	Groupwork (GEM-27)
Crane and Eckhardt (2013); USA	Quant (*n* = 82)	Not reported	26 Weeks	Not reported	Not reported	No	N/A
Crane et al. (2015); USA	Quant (*n* = 60)	Not reported	Not reported	Not reported	Not reported	No	N/A
Cuevas and Bui (2016); USA	Quant (*n* = 180)	Duluth	52 weekly 2-hr sessions	Could miss three sessions	46% (*n* = 83)	No	N/A
Cunha et al. (2022); Portugal	Quant (*n* = 83)	CBT and psycho-educational	18 weekly 90–120 min sessions	Could miss 25% of sessions	57.8% (*n* = 48)	No	N/A
Daly et al. (2001); USA	Quant (*n* = 220)	Feminist psycho-educational and CBT	1 intake, 1 evaluation, and 18 × 2-hr sessions (4.5 months)	Measured “attendance” rather than completion/non-completion	13–17 sessions: 11% (*n* = 24), 18+ sessions: 37% (*n* = 81)	Yes	Staff rated behavioral indicators of participation (e.g., self-disclosure)
Davis et al. (2021); USA	Qual (*n* = 16)	Faith-based	Open-ended	N/A	Not reported	No	Men’s reasons for voluntarily engaging
Davis et al. (2020); USA	Qual (*n* = 18)	CBT, trauma-informed care, and MI.	Open-ended	N/A	Not reported	No	Men’s perspectives on why program is “engaging.”
DeHart et al. (1999); USA	Quant (*n* = 61)	Not reported	12 weekly 1.5-hr sessions	Attended 12 sessions	49.2% (*n* = 30)	No	N/A
DeMaris (1989); USA	Quant (*n* = 295)	Not reported	12 sessions	Could miss one session	73.1% (*n* = 166)	No	N/A
Duplantis et al. (2006); USA	Quant (*n* = 313)	Feminist-informed CBT	24 weekly 2-hr sessions	Measured hours attended	Not reported	No	N/A
Echeburúa et al. (2006); Spain	Quant (*n* = 52)	CBT	20 weekly 2-hr sessions	Unclear	92% (*n* = 48)	No	N/A
Eckhardt and Crane (2014); USA	Quant (*n* = 26)	Not reported	Not reported	Unclear. Measured “compliance” rather than completion.	46.2% (*n* = 12; “compliant” at 6-month follow-up)	No	N/A
Eckhardt, Holtzworth-Munroe, et al. (2008), Eckhardt, Samper, et al. (2008); USA	Quant (*n* = 199)	Not reported	24 Weekly sessions	Could miss three sessions, had to attend exit interview	59.1% (*n* = 118)	Yes	N/A
Gerlock (2001); USA	Quant (*n* = 62)	Duluth	26 (minimum) weekly 2-hr sessions	Open-ended: defined as transitioning from rehabilitation to maintenance	37% (*n* = 23), completers attended an average of 37 sessions	Yes	N/A
Gondolf (1990); USA	Quant (30 providers)	Multiple programs (incl. psycho-educational, and didactic/confrontational)	Multiple program lengths	Not reported	38% of providers reported completion rates over 75%	No	N/A
Gondolf (2000); USA	Quant (*n* = 994)	Gender-based cognitive-behavioral treatment	15 Weekly 1.5-hr sessions	Dismissal after two missed sessions	1994: 74% 1995: 78% 1997: 69%	No	N/A
Gondolf (2008a); USA	Quant (*n* = 501)	Gender-based cognitive-behavioral treatment	16 Weekly 1.5-hr sessions	Could miss two sessions	52%—55% (across six program conditions)	No	N/A
Gondolf (2008b); USA	Quant (*n* = 684)	Gender-based, cognitive-behavioral curriculum	16 weekly 1.5-hr sessions	Must complete all sessions	54.1% (*n* = 370)	Yes	N/A
Gondolf (2009); USA	Quant (*n* = 479)	Gender-based, cognitive-behavioral curriculum	16 weekly 1.5-hr sessions	Completion defined as not having been dismissed by program staff	57% (*n* = 273)	Yes	N/A
Gondolf and Foster (1991); USA	Quant (*n* = 200)	Psycho-social education	32 Weekly sessions	Unclear	7% (*n* = 13) of inquirers attended 12+ sessions	No	N/A
Gray et al. (2016); Australia	Qual (*n* = 21)	Not reported	18 Weekly sessions	Not reported	Not reported	Yes	Impact of perceptions of relationship on motivation to attend.
Gray et al. (2014); Australia	Qual (*n* = 24)	Three programs all draw on Duluth.	Varied (6 weeks + 18 × 3-hr weekly sessions; 15 weeks; 18 weeks)	Not reported	Not reported	Yes	Men’s perceptions of group dynamics and peer relationships.
Grusznski and Carrillo (1988); USA	Quant (*n* = 175)	Structured group therapy, cognitive-based program.	32 × 2.5-hr sessions, initially twice a week, then once a week	Attending at least 14 of the 16 sessions in phase 2.	43.7% (*n* = 59)	No	N/A
Hamberger et al. (2000); USA	Quant (*n* = 482)	CBT	16 weeks (3 individual sessions, 12 × 2-hr group sessions and one review session).	Complete 3 assessment sessions and 12 treatment sessions. Clients who miss final review still considered completers.	57.1% (*n* = 275).	No	N/A
Hamel et al. (2022); USA	Quant (*n* = 175)	Five programs (mainly client-focused CBT)	Not reported	Not reported	Not reported	No	Discusses factors that promote engagement
Heckert and Gondolf (2000); USA	Quant (*n* = 840)	Three programs, all CBT	Program 1: 3 months Program 2: 3 months Program 3: 5.5 months	Between 12 and 36 sessions	Not reported	Yes	N/A
Huss and Ralston (2008); USA	Quant (*n* = 175)	Duluth and CBT	Not reported	Not reported	54.9% (*n* = 96).	No	N/A
Lauch et al. (2017); USA	Quant (*n* = 202)	CBT and feminist-informed	25 weekly 1-hr sessions (incl. intake)	Attend all sessions for the majority of their duration.	50.5% (*n* = 102).	No	N/A
Levesque et al. (2012); USA	Quant (*n* = 492)	Psychoeducational; transtheoretical model	40 hr over 20 weeks	Not clearly defined, relies on info from agency staff.	68.1% (*n* = 323)	Yes	N/A
Lila et al. (2020); Spain	Quant (*n* = 286)	CBT	30–32 weekly 2-hr sessions	Not clearly defined	72.0% (*n* = 206)	No	N/A
Maldonado and Murphy (2021); USA	Quant (*n* = 195)	CBT	20 weekly 2-hr sessions	Not reported	Not reported	No	Homework compliance, working alliance
Morrison, Cluss, et al. (2019), Morrison, George, et al. (2019); USA	Qual (*n* = 76)	Varied (data from 2 programs): combined CBT and educational approach; Duluth	Varied: 16 and 24 weeks	Not reported	Not reported	No	Group process and dynamics, the role of facilitators
Morrison, Jones, et al. (2019); USA	Quant (*n* = 330)	Combined CBT and educational approach	24 Weeks	Attended all sessions mandated by the court	63.1% (*n* = 188)	No	N/A
Munro and Sellbom (2022 ); USA	Quant (*n* = 531)	CBT and Duluth.	24 × 1-hr sessions conducted over 6, 12, or 24 weeks	Could miss two sessions, had to follow program rules, and pass exam on program content	63.3%	No	Participation rating, participation variability.
Musser et al. (2008), Murphy et al. (2012) (uses subset ; *n* = 83) from Musser et al. (2008); USA	Quant (*n* = 108 [MI *n* = 55, control *n* = 53])	CBT. Pre-treatment MI sessions or standard intake sessions ×2	16 sessions	Not reported	Not reported	No	Working alliance, homework compliance, attendance, other help-seeking, in-group behavior
Parra-Cardona et al. (2013); USA	Qual (*n* = 21)	Culturally informed version of the Spanish version of Duluth	26 weekly 2-hr sessions	Not reported	Not reported	No	Awareness of violent behaviors, accountability, change in actions, relationship with leaders, inter alia
Poole and Murphy (2019); USA	Quant (*n* = 210)	CBT	20 weekly 2-hr sessions	Could miss two sessions	61.4% (*n* = 129)	No	Attendance, homework compliance, readiness to change, working alliance, inter alia
Priester et al. (2019); USA	Quant (*n* = 268)	Psychoeducational	26 weeks	Up to four “excused” absences permitted.	33.2% (*n* = 89)	No	N/A
Rock et al. (2013); USA	Quant (*n* = 483)	Duluth and CBT	Varied: 6, 12, and 24 weeks	Could miss 1 out of 6, 2 out of 12.	65.4% (*n* = 293; *data available for 92.8% of sample.)	No	N/A
Romero-Martínez et al. (2021); Spain	Quant (*n* = 424)	CBT; motivational strategies	35 weekly 2-hr sessions	Not clearly defined.	83.5% (*n* = 354)	No	N/A
Romero-Martínez et al. (2023a); Spain	Quant (*n* = 561)	Psychoeducational	Approx. 35 weeks	Not clearly defined.	78.1% (*n* = 438)	No	N/A
Romero-Martínez et al. (2023b); Spain	Quant (*n* = 427; perps *n* = 324, control *n* = 103)	CBT; motivational strategies	35 weekly 2-hr sessions	Not clearly defined.	88.6% of perpetrator sample (*n* = 287)	No	N/A
Rondeau et al. (2001); Canada	Quant (*n* = 286)	Varied (Data from 8 programs).	Varied: 14–15, 20–21, 25 weeks	“Remaining in the basic program till its end”	37.3% (*n* = 196)	No	N/A
Rooney and Hanson (2001); Canada	Quant (*n* = 306)	Varied (Data from 4 programs).	Varied: 12, 14, 18, and 25 sessions.	Not clearly defined.	50.0% (*n* = 153)	Yes	N/A
Rothman et al. (2007); USA	Quant (*n* = 3,460)	Varied (data from 26 programs).	Not reported	Classified as “complete” or “noncomplete” by counselors.	40.2% (n = 1,391)	No	N/A
Roy et al. (2013, 2014); Canada	Qual (*n* = 40)	Program 1: CBT and feminist, Program 2: CBT and psychodynamic	25 sessions	Attend 20 sessions	Not reported	No	Attendance, contribution, relating to worker and other members, inter alia
K. L. Scott (2004); Canada	Quant (*n* = 308)	Feminist-informed, Duluth	17 weeks	Attend all sessions	38.6% (*n* = 119)	No	N/A
K. Scott et al. (2011, 2013) (uses subset; *n* = 294) from K. Scott et al. (2011); Canada	Quant (*n* = 486)	Duluth-style (control group); Duluth and MET (experimental group)	16 weeks	Could miss up to two sessions. File closure due to new criminal charges also resulted in an incomplete classification.	59.9% (*n* = 276)	No	Counselor-rated success (achievement of goals focused on active engagement and accountability)
Stanley et al. (2012); UK	Mixed method (admin data, *n* = 32; qual *n* = 21)	Participant-led and staff-led structured discussion.^b^	10 individual sessions, and 1-year group program.	“Engaged participants”: completed 5+ individual sessions in the first 6 months.	46.9% (*n* = 15; Proportion of “engaged” participants)	Yes	“Sustained commitment” to attendance.
Stosny (1994); USA	Quant (*n* = 106 [video *n* = 54; control *n* = 52])	Variations of feminist, CBT, family systems and integrative approaches. Pre-treatment MET.	Full treatment length not reported	Attend at least three-quarters of scheduled sessions.	85.8% (*n* = 91) Video (94.4% *n* = 51), standard (76.9% *n* = 40).^c^	No	Initiated group response, homework fee payment, inter alia
Taft, Murphy, Elliott, and Keaser (2001); USA	Quant (*n* = 101)	CBT	16 weekly 2-hr sessions	Attend at least 75% of scheduled sessions	69.3% (*n* = 70)	No	N/A
Taft, Murphy, Elliott, and Morrel (2001); USA	Quant (*n* = 189 [control *n* = 106; retention, *n* = 83])	CBT (control); CBT or supportive therapy (treatment); MET retention (treatment)	16 weekly 2-hr sessions	Attend at least 75% of scheduled sessions	76.7% (*n* = 145) Retention treatment: 85.5%, *n* = 71 Control group: 69.8%, *n* = 74	Yes	N/A
Ting et al. (2009); Country not reported	Quant (*n* = 145)	CBT	16 weekly 2-hr sessions	“Credible dose,” attending 75% or more of sessions.	69.0% (*n* = 98).^d^	Yes	Homework, group cohesion, working alliance, inter alia
Tollefson and Phillips (2015); USA	Quant (*n* = 90 (MBB *n* = 44; control *n* = 46))	MBB (experimental) and psychoeducational (control)	16 × 60-min sessions within (MBB) 20 × 60-min sessions (control)	Not clearly defined.	83.3% (*n* = 75) MBB (*n* = 40, 90.9%), control (*n* = 35, 76.1%).^e^	No	N/A
Trebow et al. (2015); USA	Mixed methods (*n* = 150)	Not reported	52 Sessions completed within 18 months	Could miss up to three sessions	68% (*n* = 102)	No	N/A
Whitman et al. (2020); USA	Quant (*n* = 478)	Duluth and CBT	Varied: 6, 12, or 24 sessions	Staggered. Could miss 2 of 6 or 3 of 12 sessions.	66.8% (*n* = 290)^f^	No	N/A

*Note*. MBB = mind-body bridging; MET = motivational enhancement therapy; MI = motivational interviewing.

a Completion data only available for 461 participants.

b Program participants were offered individual sessions informed by cognitive approaches, counseling, behavioral techniques, and elements of the mindfulness approach prior to group work.

c Stosny (1994) used “attending meetings regularly” variable.

d Completion data reported only for the 142 participants who completed the Alcohol Use Disorders Identification Test and Drug Abuse Screening Test.

e Tollefson and Phillips (2015) report non-completion rate of 16 of 90 (16.7%), however 16/90 = 17.7%. Completion rate reported here therefore assumes non-completion of 15/90 (16.7%).

f Completion data available for 90.8% (*n* = 434) of final sample.

### Study Characteristics

The included sources were published between 1988 and 2023. Most studies were conducted in the United States (*n* = 58, 72.5%), followed by Canada (*n* = 9, 11.3%), Spain (*n* = 6, 7.5%), Australia (*n* = 2, 2.5%), United Kingdom (*n* = 2, 2.5%), and Portugal (*n* = 1, 1.3%). One study did not report location. Most articles used quantitative methods (*n* = 63, 78.8%), followed by qualitative methods (*n* = 13, 16.3%) and mixed methods (*n* = 4, 5.0%). The sample sizes ranged from 6 to 3,460. Most interventions employed cognitive-behavioral therapy and/or Duluth approaches, although program approach was often vaguely described. Length of programs varied from 6 to 52 weeks, or in some cases was “open-ended,” though program length was unclear in 10 studies. Thirteen sources (16.3%) incorporated victim-survivor data, however, these data were largely used to assess men’s use of violence or validate men’s reports of change, with only one using it to understand men’s program engagement (Gray et al., 2016). A nuanced consideration of engagement beyond attrition was evident in 25 (31.3%) studies. Of these, 11 (44.0%) were quantitative, 12 (48.0%) were qualitative, and 2 (8.0%) used mixed methods.

## Discussion

### Program Completion

Consistent with Jewell and Wormith (2010), we found completion rates varied substantially, ranging from 7% to 92%, with an average of 53.8% (*SD* = 19.9). Such disparity reflects, in part, the varied and arbitrary definitions used across studies (see also Babcock et al., 2004). For example, some studies defined completion as a requirement for participants to attend *all* sessions, or to miss up to one, two, three, or four sessions (see Table 2). In practice, this could vary from a participant missing 2 out of 16 sessions, 2 out of 32 sessions, missing 3 out of 24 sessions, or 3 out of 52 sessions. In Rock et al. (2013), the requirement was staggered, allowing participants to miss 1 out of 6, or 2 out of 12 sessions. A few studies opted for a percentage-based definition, whereby participants could miss 25% of the total program offerings.

Recognizing the arbitrariness of completion, some studies moved away from a dichotomous completion variable and instead used the number of sessions attended as the dependent variable in their analyses (Duplantis et al., 2006; Maldonado & Murphy, 2021; Murphy et al., 2012). Some studies did not include clear definitions of completion (Chovanec, 2012; Echeburúa et al., 2006; Gondolf, 1990; Huss & Ralston, 2008), and others provided only vague definitions, such as “left the intervention before it ended” (Romero-Martinez et al., 2021, p. 321, 2023a, 2023b). The way completion was measured also varied, with some studies counting sessions from initial inquiries and others from program start date.

In some programs, “completion” entailed additional requirements such as adhering to the program rules and completing a final exam (Munro & Sellbom, 2022), or an exit interview (Eckhardt, Holtzworth-Munroe, et al., 2008). Other definitions were based on whether program staff defined participants as completers (Bowen & Gilchrist, 2006; Levesque et al., 2012; Rothman et al., 2007) or did not dismiss them (Gondolf, 2009), or whether the participant fulfilled the magistrate’s mandate (Morrison, George, et al., 2019). Interestingly, Bowen and Gilchrist (2006) adopted the “whether program staff defined participants as completers” approach because they identified discrepancies in practitioners’ understanding of the number of absences that amounted to non-completion (despite this approach arguably introducing *more* scope for error or bias). Regardless of the approach, few studies offer clear and consistent definitions of or justifications for what is measured. Yet, completion remains a key outcome utilized across studies.

### Factors Influencing Completion: Mapping Messiness

Most studies captured in this review (*n* = 73, 91.3%) examined the impact of risk/protective factors on completion (variously defined). Several reviews offer analyses of such associations (Daly & Pelowski, 2000; Jewell & Wormight, 2010; Olver et al., 2011), and we do not intend to repeat this work. Rather, we conduct a brief content analysis of the breadth of variables assessed and their impact on completion to illustrate the messiness of establishing “risk,” and the need to consider how structural inequities shape capacity to complete.

In most studies, program completers were older (*n* = 16, 66.7%), more highly educated (*n* = 7, 46.7%), employed (*n* = 10, 55.6%), and/or earning a higher income (*n* = 10, 58.8%) than non-completers. Several studies found completers were more likely to have “stable” family lives, and less likely to have criminal/arrest histories or substance use problems. However, in relation to other “risks,” the identified factor may be misleading or signify the effect of a different factor. For example, several studies found participants from minority ethnic groups were less likely to complete, however, Bennett et al. (2010) note that the effect of race on completion disappeared when controlling for class. An association between ethnicity and program completion may thus more accurately reflect inequalities in access to resources.

Studies also examine the effect of referral status (e.g., court-mandated), offender typology, and psychological/neurological characteristics on program completion. Of particular interest, eight studies (66.7%) found that court mandates increased the likelihood of program completion (while four reported it as insignificant). However, little is known about differences in the quality of engagement or changes in use of violence among those who are mandated compared to those who enter via other referral pathways (Fitz-Gibbon et al., 2024). Research that measured the effect of psychopathology variously reported that non-completers were more likely to have psychopathological traits such as PTSD (Gerlock, 2001) or borderline personality disorder (Munro & Sellbom, 2022), exhibit antisocial traits (Chang & Saunders, 2002; Rock et al., 2013), score high on scales such as the State–Trait Anger Expression Inventory (Eckhardt, Samper, et al., 2008), or that measures of psychopathology did not have a significant effect (*n* = 8, 57.1%; e.g., Buttell & Carney, 2008; Gerlock, 2001). Relatedly, Romero-Martinez et al. (2021, 2023a, 2023b) found that neurodivergence and “low” neurological performance were associated with non-completion, though, notably, few other studies investigate this relationship. Other factors of interest were whether program completers had experienced abuse in their family of origin, and what type of violence they had perpetrated before program participation. For example, several studies found that participants who exited early were more likely to have experienced violence in their family of origin (*n* = 4, 50.0%), and Carney et al. (2006) found that non-completers were more likely to have used sexual coercion tactics.

Taken together, we can see that there are patterns in risk factors for non-completion. However, it is critical to consider how we interpret and work with these patterns. That is, rather than individualizing perceived risks such as low education levels, there is a need to consider what supports are required to increase engagement for such participants. Furthermore, there are notable exceptions for these patterns across studies in which no significant relationship or an inverse relationship is found. This may be due to small sample sizes, or the misclassification of risk factors (e.g., through misreporting of salary size). Indeed, the conflicting results reported across this review could partially reflect methodological differences, but they also speak to individual differences among participants and the need for a more in-depth understanding of program engagement beyond completion.

### Studies of Engagement

Of the 80 sources included in this review, 25 (31.25%) examined program “engagement” beyond attrition. Across these studies, engagement was understood and operationalized in terms of group cohesion, working alliance and relationship with facilitators, group contribution and participation, resistance to attendance and engagement, motivation, and belief in the value of group. These components were examined using quantitative measures including the working alliance inventory (WAI), the group engagement measure, factors such as homework compliance, and other forms of in-depth, qualitative data.

### Group Cohesion

Participants across the included studies spoke about the value of bonding with other program participants, building rapport, trust, and mutual respect, and the role of mentoring and support between group members (Bouchard & Wong, 2021; Davis et al., 2020; Gray et al., 2014; Morrison, George, et al., 2019; Roy et al., 2014). Some participants specifically associated the supportive group environment with making them “want to change” (Bouchard & Wong, 2021, p. 2651) and spoke about gaining insights into their own abusive behavior by listening to and learning from others’ experiences (Davis et al., 2020; Gray et al., 2014; Morrison, George, et al., 2019). The peer environment was also considered useful for challenging abusive behaviors. As one participant explains, “Sometimes it is hard taking a look at yourself, somebody calling you out on your s**t. You realize ‘Hey, maybe there are some things that I’m doing wrong,’ and so you can get a lot out of that” (Morrison, George, et al., 2019, p. 1884).

Conversely, negative peer interactions could undermine group cohesion, as engagement became “inauthentic” (Gray et al., 2014, p. 396). Participants described a range of interactions that interfered with the group dynamic. This included domineering, disruptive, and resistant participants, those who lied or denied having a problem, were disinterested or indifferent to other group members, or did not contribute (Morrison, George, et al., 2019). While a cohesive group is an indicator of engagement, critically such “inauthentic” or pseudo-engagement, where for example, interactions during break are inconsistent with presentations within group sessions, may not be visible to program staff or evaluators (Renehan, 2021).

It was common across studies for participant feedback to say that there was a “great group of guys” and that they valued “supporting each other” (Bouchard & Wong, 2021, p. 2651; Chovanec, 2012; Gray et al., 2014; Parra-Cardona et al., 2013; Roy et al., 2013). Furthermore, participants spoke about the value of “knowing and hearing I’m not alone *suffering* from this situation” (Bouchard & Wong, 2021, p. 2652, emphasis added) and reflected that the group program was one of the few places they felt they could talk about their problems (Morrison, George, et al., 2019). Yet, the “authenticity” or quality of engagement was sometimes unclear. For example, while the participant quote above speaks to the value of shared experiences and knowing they are “not alone,” the focus on their own “suffering” could indicate ongoing adherence to a victim stance. Concerns about genuine engagement have also been raised by victim-survivors. For example, a victim-survivor in Gray et al.’s (2016, p. 177) study commented “I don’t think he was absolutely, totally genuine. [. . .] You can attend university but if you don’t study, you won’t pass sort of thing, you know?”

### Working Alliance

Participants’ perception of and respect for facilitators, their expertise and experience, also appeared to impact engagement (Hamel et al., 2022; Morrison, Cluss, et al., 2019; Roy et al., 2013). Some studies report that participants’ perception of the group experience tends to be more favorable where facilitators are perceived as caring, committed, non-judgmental, humble, authentic, and able to create a safe group environment where participants are treated “as equals” (Bouchard & Wong, 2021, p. 2652; Hamel et al., 2022; Morrison, Cluss, et al., 2019). Furthermore, Morrison, George, et al. (2019, p. 490) found participants’ perception of facilitators as invested in the intervention, “its mission” and their clients, to influence engagement.

Quantitative assessments of participants’ relationship with facilitators often utilize a form of the WAI (Horvath & Greenberg, 1986; Tracey & Kokotovic, 1989). Among these studies, factors including borderline and antisocial characteristics (Maldonado & Murphy, 2021), and substance abuse (Ting et al., 2009) have been associated with weaker working alliance. For example, Ting et al. (2009) found that substance abuse was associated with lower scores on WAI items including agreement on the goals/tasks of therapy, emotional connection, and engagement with group facilitators and other group members. Maldonado and Murphy (2021) found readiness to change to be positively associated with working alliance, noting that predictive association between readiness and working alliance was moderated by borderline and antisocial characteristics and court referral status. Interestingly, Poole and Murphy (2019) found a marginally statistically significant difference in therapist-reported WAI scores later in treatment, such that they were higher for fathers than non-fathers (60.7 vs. 56.3, *p* = .08), though men’s self-report WAI scores were similar between fathers and non-fathers.

### Contribution

Contribution, a key element of Macgowan’s (2006) model of engagement, was discussed in many studies included in this review (Morrison, George, et al., 2019; Roy et al., 2014; Stosyny, 1994). Though related to “cohesion”—a group-level phenomenon that describes the quality of relationships—contribution refers to participants’ *individual* participation and input. Specifically, participants across the studies in this review valued hearing and learning from other group members’ experiences, and likewise seeing how others benefited when they shared their own stories and advice. As one participant explained, “I like helping the other men out, especially when I feel like I can really speak about some of my experience and actually say I did this and this worked for me” (Morrison, George, et al., 2019, p. 1886). Crucially, however, giving advice does not in itself indicate meaningful engagement because in isolation it may serve to deflect accountability for one’s own use of violence (Roy et al., 2014). Illustrating the relational nature of the components of engagement, contribution must be accompanied by other practices (such as working on one’s own problems) and a broader motivation to change. It is worth noting, though, that the quality of participants’ contribution may be influenced by factors such as being introverted (Roy et al., 2013), traits related to borderline personality disorder (Munro & Sellbom, 2022), and competing demands such as work (Bouchard & Wong, 2021). For example, one participant reflected, “sometimes you are drained from the day or other appointments and have a hard time concentrating” (p. 2654).

Related to the previous discussion about the role of facilitators, participants in Morrison, Cluss, et al.’s (2019) study felt that the gender of the facilitator(s) influenced their contribution. Some men reported feeling that they could express themselves more with a woman facilitator. Similarly, others felt that *without* a woman facilitator, groups may be “one-sided” (p. 497) and having a one-man-one-woman co-facilitator model offered “both points of view” as “you can see what the male version looks like, and the female version” (p. 496). Yet this illustrates how participants’ interactions with facilitators and contributions to the group can reflect and reinforce gender norms (that, e.g., emotion work is women’s work), thereby counteracting broader program aims of gender transformative change. Understanding these dynamics is crucial to situating assessments of engagement within broader gender structures.

One study included in our analysis offered insights into how delivery format may influence contribution. Unsurprisingly, online programs are useful when location is a barrier to participants’ attendance and completion (see Bouchard & Wong, 2021). Beyond this, facilitators and participants in Bellini and Westmarland’s (2021, p. 507) study said building trust was “a lot easier” online, due to increased privacy and the familiarity of being “in a place that you know.” Likewise, facilitators reported higher levels of attendance compared to in-person programs they had facilitated, as well as greater contribution and openness. We note however, that research in this area remains limited, and that additional barriers such as internet, software, and hardware accessibility, as well as technology literacy, may impede access to online programs (Bellini & Westmarland, 2021).

### Early Resistance to Attendance and Engagement

Initial reluctance and resistance to attend group sessions were commonly described across studies. At a logistical level, initial reluctance is exacerbated by long waitlists, with some men seeking help but feeling “discouraged and alone” while waiting for group to start (Roy et al., 2013, p. 1808). Describing how their early resistance manifested, one participant said, “I’d show up, take a seat [. . .] cross my arms, and act as if I didn’t want to know anything” (Roy et al., 2014, p. 428). However, positive peer interactions and the realization that one is not alone was said to alleviate initial reluctance and promote engagement (Gray et al., 2014, p. 397; Morrison, George, et al., 2019). To this end, ensuring content is relatable is also crucial, as participants noted that “irrelevant” topics hindered their engagement. For example, one participant said that they had a “good loving family growing up,” and therefore found discussions of childhood abuse “hard to relate to” (Bouchard & Wong, 2021, p. 2654). This could indicate the value of tailored groups that address specific factors, such as the use of AODs, fathering, or childhood trauma. However, perceptions of topic or program “irrelevance” may also indicate the denial of violence perpetration.

Some studies (Parra-Cardona et al., 2013; Stosny, 1994) found videos and vignettes useful for establishing relatability. For example, Stosny (1994) examined the use of a video portraying a treatment-resistant client and a domestic violence scene. The video is intended to reflect program participants’ feelings of anger and humiliation (as they relate to the “client” in the video) and then instill compassion through consideration of the impact on the mother, child, and perpetrator. The video, followed by group discussion, was found to increase engagement as measured through attendance the following week, staying after the session for further discussion, requesting the handout with additional information, and increased contribution. Some studies speak of a “turning point” (Roy et al., 2013, p. 1807) at which participants’ resistance abates and they begin to engage more meaningfully. For example, the participant quoted above who spoke of his initial resistance reflected on how this changed partway through the program and as they describe, “after five or six meetings, something clicked in my mind [. . .] I unfolded my arms and I began to open up” (Roy et al., 2014, p. 428). While this is sometimes framed as a “light bulb” moment, Kelly and Westmarland (2015, p. 34) argue this phrase does not reflect the “require[d] layers of understanding, reflection and translation into behavior,” and “[c]hange is better understood as a series of sparks, different for each man, and not all of which are activated.”

### Motivational Interviewing and Readiness to Change

One intervention found to influence various components of engagement is motivational interviewing (MI). For example, Musser et al. (2008) found both client and therapist reports of working alliance to be enhanced by MI. They also found MI as a pre-treatment intervention was associated with constructive behavior, such as responses toward others during early group sessions, and greater homework compliance among participants who undertook motivational intake processes. However, the usefulness of MI for engagement may not be the same for all participant groups. Murphy et al.’s (2012) research indicates that MI is most useful for participants who are in early stages of change, have high trait anger and are more treatment resistant (see also Musser et al., 2008). Notably, the nature and extent of MI treatment, including whether this is a pre-treatment intervention or embedded into the main intervention, vary across studies.

Additionally, Musser et al. (2008) found MI procedures at intake were associated with greater uptake of available supports provided outside of the core program delivery, which may indicate deeper engagement in the process of change. Though they did not explore the effect of MI specifically, it is worth noting here that other studies also highlight men’s desire for support beyond the group program (Parra-Cardona et al., 2013; Roy et al., 2013). This includes support for co-occurring needs such as substance abuse (Ting et al., 2009) and psychological distress (Roy et al., 2013). Importantly, in programs where additional support was available, for example, meeting with facilitators outside of group for individual support, this provision was framed as an “exceptional measure” (Roy et al., 2013, p. 1810).

### Motivation: Fatherhood and Reconciliation

Motivation, particularly intrinsic motivation, is critical for participants’ meaningful program engagement. An example of an intrinsic motivation is to build a better relationship with one’s children, while an extrinsic motivator would be to regain access to one’s children or to have their child protection case closed. Stanley et al. (2012) argue that while both types of motivation can be useful for securing men’s initial attendance, intrinsic motivations may be correlated with better outcomes. Fatherhood and a desire for reconciliation stand out as two key motivating circumstances within the reviewed sources. In terms of the former, program participants describe a desire to be a “better father” (Stanley et al., 2012, p. 270; see also Roy et al., 2013; Poole & Murphy, 2019), as is recognized in domestic violence program interventions tailored to fathers (Diemer, 2020; Hine et al., 2022; Meyer, 2017). Fear of separation or a desire for reconciliation also emerged as a common factor in men’s initial help-seeking and program attendance (Davis et al., 2020; Gray et al., 2016; Rondeau et al., 2001; Stanley et al., 2012), and their readiness to change (Maldonado & Murphy, 2021). For participants who frame their attendance as being *for* their partners, separation may decrease attendance and/or meaningful engagement. For example, a participant in Roy et al.’s (2013, p. 1807) study stated “Let’s say my girlfriend had dropped me. I wouldn’t be here now. Get serious, I would’ve been out of here! I wouldn’t have busted my ass to try this therapy.” Rondeau et al. (2001) also report a correlation between an (ex)partner leaving and program dropout, suggesting some men’s motivation is solely rooted in an extrinsic desire to maintain their relationship.

Relatedly, through phone interviews with program participants and their current or former partners, Gray et al. (2016) found that (ex)partners’ perceptions of men’s engagement in the program impact participants’ motivation. Specifically, when partners question men’s commitment to the program or make a “negative appraisal of the sustainability” of their behavior change, participants experienced reduced motivation and feelings of “frustration and hopelessness” (Gray et al., 2016, p. 177). Likewise, the authors found that relationship separation can negatively impact “motivation and behavior change processes.” While specific circumstances may be valuable for tailoring interventions and enhancing motivations, where men’s engagement depends on their partners’ reactions, staying in the relationship, or the hope of reconciliation, this undermines attempts to shift the burden of responsibility away from victim-survivors. There is a need to be attentive to the nature of these motivations and to work with men “to broaden their motivations” (2016, p. 171) beyond a dependence on the actions of others.

### Belief in the Value of Group

Participants’ belief in the value and usefulness of the group emerged as an important engagement factor. This notion of “value” was described in relation to learning and behavior change; that, for example, participants were motivated to continue attending if they felt they were “learning things,” such as practical tools for emotional regulation (Chovanec, 2014; see also, Bouchard & Wong, 2021; Davis et al., 2021; Parra-Cardona et al., 2013). “Learning” from the experiences of other group members has also been linked to positive peer interactions and group cohesion (Davis et al., 2020; Gray et al., 2014; Morrison, George, et al., 2019). Participants’ perception of behavior change also appears to promote engagement. Bouchard and Wong’s (2021) participants expressed that seeing changes within themselves motivated them to continue to engage in the program. Similarly, hearing other participants’ stories of success was described as a source of motivation, as it instills the idea that change is possible (Chovanec, 2014; Morrison, George, et al., 2019). In addition to group value, individuals’ sense of value, pride, and self-worth have been found to influence engagement. As expressed by one participant “if you respect yourself, you commit” (Roy et al., 2013, p. 1806).

## Limitations

This study is subject to several limitations. First, only English language papers were included. Second, unpublished gray literature was excluded. Third, U.S. research dominates the sample, and all studies are from the Global North. Fourth, we did not test the predictive strength of variables on completion or engagement. Given these limitations, it is worth emphasizing that the findings of this systematic review primarily reflect research conducted in Western, English-speaking countries. Additionally, most studies included do not report on participants’ race, ethnicity, or linguistic backgrounds, and few account for intersecting identities such as sexuality or disability. Where variables such as socioeconomic status and referral status were reported, we have considered how these factors may interact with engagement and completion. For example, we note how associations between ethnicity and non-completion may in fact reflect a lack of access to resources, and that court-mandates may improve completion rates, but little is known about the specific impact on engagement. However, these variables are often treated as static risk factors rather than dynamic social characteristics that are situated within broader structural inequities. This limits the depth and generalizability of our discussion, and our ability to assess how engagement in perpetrator programs is shaped by intersecting identities, systemic barriers, and diverse lived experiences. Future research should thus seek these deeper intersectional understandings. These limitations notwithstanding, this study consolidates the substantial research on program completion and engagement, offering a unique contribution to broader discussions regarding how we understand, measure, and examine domestic violence perpetrator program outcomes and effectiveness.

## Conclusion

Our findings demonstrate a continued reliance on completion and recidivism outcomes as indicators of domestic violence perpetrator program effectiveness. Yet these may not be meaningful indicators of behavior change. This article illustrates that definitions of completion used in the field are arbitrary and inconsistent and may not translate into violence reduction. Participants who engage meaningfully in a program but exit early due, for example, to broader structural inequities such as access to housing, may take away greater learnings and reductions in violence than a participant who attends a full program to comply with court orders but does not meaningfully engage (see also, Fitz-Gibbon et al., 2024). When the true outcome of interest is violence cessation, we must question the usefulness of completion and recidivism as proxies for assessing change.

Engagement is not always predictive of program completion and warrants unique examination. Our analysis shows that within the landscape of domestic violence perpetrator intervention research, examinations of engagement are few. In examining this literature, we highlight the need for a more nuanced approach to measuring engagement that captures its multi-dimensional nature. Assessing individual factors of engagement in isolation or focusing solely on behavioral indicators (such as showing up or offering advice) can result in limited and inaccurate assessments. Programs and their evaluators should assess a broad range of dimensions including emotional engagement (e.g., trust, commitment and motivation), cognitive engagement (e.g., taking on program learnings), relational engagement (e.g., connecting with peers and facilitators), and contractual engagement (e.g., belief in the value of the program), as well as accounting for contextual dimensions (e.g., access and cultural safety) that enable or constrain engagement. Additionally, our review findings demonstrate the relational and non-linear nature of program engagement and behavior change more broadly; that participants’ engagement may be interspersed with periods of apathy or disengagement. This again highlights the need for program providers and evaluators to move away from a focus on “static” factors and instead design evaluation frameworks that capture the complex, fluid, and at times contradictory nature of how people who use domestic violence engage in change via program attendance and participation.

These changes ultimately rely on broader shifts to program funding and compliance processes. First and foremost, program funding needs to facilitate the full breadth of work required by providers to engage participants—including but not limited to program readiness work, motivational work and resources required to meaningfully address structural barriers, where possible. Alongside this, we recommend a move away from contractual reporting and compliance requirements that use completion as a core metric, and instead integrate more flexible frameworks for providers. We recognize that complex forms of evaluation data may not fit neatly within existing reporting expectations and will thus require a substantial shift in current funding reporting requirements. However, it may facilitate more accurate reporting of effectiveness; that is, whether providers are achieving the kind of engagement that best supports program participants’ reduction in and desistance from domestic violence. Finally, given some engagement opportunities occur outside of perpetrator programs through other points of system contact (e.g., child protection and courts), we recommend greater integration across services, to support and reinforce the work of behavior change programs, where possible.

**Table:** Summary of Critical Findings.

Finding	Description
Defining/measuring completion	Completion rates varied substantially (from 7% to 92%), reflecting, in part, the inconsistent and arbitrary definition of completion utilized across studies.
Risk/protective factors	There are some patterns in risk/protective factors for completion. However, the breadth of variables assessed and the inconsistency in their effect illustrate the messiness of establishing “risk,” and the need for a more in-depth understanding of program engagement that moves beyond a binary measure of completion/non-completion, and accounts for structural inequities.
Factors that promote “engagement”	In the landscape of domestic violence perpetrator intervention research, examinations of engagement are few, but key findings in this literature include: *Group cohesion*: Participants emphasized the value of bonding with other participants, building rapport, trust, and mutual respect, and the role of mentoring and support between group members. *Working alliance*: Participants’ perception of the group experience tends to be more favorable when facilitators are perceived as caring, committed, non-judgmental, humble, authentic and able to provide a safe group environment where participants are treated “as equals.” *Contribution*: Participants valued hearing and learning from other group members’ experiences, as well as being able to help each other through sharing their experiences. *Overcoming early resistance*: Initial reluctance to attend group sessions was commonly described, and said to be exacerbated by long waitlists. Ensuring content is relatable and leveraging shared experiences between group members can help overcome this initial resistance. *Motivational interviewing*: Several studies found motivational interviewing enhanced engagement by promoting working alliance, homework compliance, constructive group behavior, and additional support uptake, though this effect varies according to participant groups. *Intrinsic motivation*: While all motivation (broadly defined) helps secure men’s initial attendance, “intrinsic” motivations may be correlated with better outcomes. Two key motivating circumstances stand out within the reviewed sources: fatherhood and a desire for reconciliation. Belief in the value of group: Participants’ belief in the value and usefulness of the group (e.g., learning practical skills, learning from others’ experiences, or seeing change in oneself) emerged as an important engagement factor.

**Table:** Implications for Practice, Policy, and Research.

Practice	• Interventions should move away from arbitrary requirements of program attendance to approaches that foster meaningful engagement. • Greater awareness and assessment of engagement as a reflexive practice could be beneficial to guide timely, tailored support to enhance engagement in DV perpetrator programs.
Policy	• Program funding needs to facilitate the full breadth of work required to engage participants—including but not limited to readiness work, motivational work and addressing structural barriers. • Contractual reporting/compliance requirements that move away from completion as a core metric creating flexibility for providers to assess more meaningful engagement that best supports program participants reduction in and desistance from violence. Recognizing that reporting this data may not fit neatly within existing reporting expectations, this requires a substantial shift of funders’ current practice. • Engagement is complex, and some engagement opportunities occur outside of perpetrator programs through other points of system contact (e.g., child protection, courts). There is a need for greater integration across services that can reinforce the work of perpetrator programs to enhance overall efforts in perpetrators’ engagement in meaningful change and ongoing desistance from violence.
Research	• Studies on DV perpetrator program completion and engagement would benefit from greater incorporation of victim-survivor perspectives. • In their design of evaluation frameworks for perpetrator programs, researchers and service providers should capture indicators of engagement beyond completion as these are critical to informing engagement strategies, and (where available) post-program support for violence desistance. This includes seeking to assess emotional, cognitive, relational, and contractual engagement as well as accounting for contextual factors in interpreting engagement.

## Data Availability

The data that support the findings of this study are available from the corresponding author upon reasonable request. A protocol for this review was registered on PROSPERO (PROSPERO 2023 CRD42023399763).

## Notes

1. Covidence systematic review software, Veritas Health Innovation, Melbourne, Australia. Available at www.covidence.org.
